# Is there publication bias towards brazilian articles on cancer?

**DOI:** 10.1590/S1679-45082013000100005

**Published:** 2013

**Authors:** Luiz Victor Maia Loureiro, Donato Callegaro, Altieres de Arruda Rocha, Bernard Lobato Prado, Taciana Sousa Mutão, Carlos del Cistia Donnarumma, Auro del Giglio

**Affiliations:** 1 Hospital Israelita Albert Einstein, São Paulo, SP, Brazil

**Keywords:** Oncology, Neoplasms, Scientific and technical publications, Publication bias

## Abstract

**Objective:**

To investigate whether Brazilian articles on cancer are published in journals with an impact factor and/or repercussion (measured by the number of citations) inferior to those that come from foreign organizations.

**Methods:**

A search was carried out in PubMed for the MeSH term “*neoplasm*” with the limits clinical trial, affiliation of the Brazilian author(s), and interval from July 1^st^, 2009 to June 30, 2010. Selected for matching were non-Brazilian related articles published from three months prior to three months after the date of publication of the Brazilian study. The numbers of citations were obtained from two databases, as well as the impact factor for the journals in which the articles were published.

**Results:**

Fortythree national and 876 related international articles were identified. The Brazilian publications had a mean impact factor of 3.000 *versus* 3.430 of the international ones (p=0.041). There was no statistically significant difference as to the number of citations between the two groups. The affiliation of the first author with a Brazilian or foreign organization did not significantly influence the number of citations or the impact factor.

**Conclusion:**

Brazilian articles are significantly less accepted in journals with higher impact factors, although it does not compromise its repercussion on the scientific community.

## RESUMO

**Objetivo:**

Investigar se artigos brasileiros sobre câncer são publicados em periódicos de fator impacto e/ou repercussão (medida pelo número de citações) inferiores aos oriundos de instituições estrangeiras

**Métodos:**

Pesquisou-se, no PubMed, o MeSH Term “*neoplasm*” com os limitadores: *clinical trial*, afiliação de autor(es) brasileira e intervalo de 1° de julho de 2009 a 30 de junho de 2010. Foram selecionados para pareamento artigos relacionados, não brasileiros, publicados entre três meses antes e três meses depois da data de publicação do estudo brasileiro. Foram obtidos os números de citações, em duas bases de dados, assim como o fator de impacto para as revistas nas quais os artigos foram publicados.

**Resultados:**

Identificaram-se 43 artigos nacionais e 876 internacionais relacionados. Os brasileiros apresentaram fator de impacto médio de 3.000 contra 3.430 dos internacionais (p=0,041). Não houve diferença estatisticamente significativa quanto ao número de citações entre os grupos. A afiliação do primeiro autor à instituição brasileira ou estrangeira também não influenciou significativamente no número de citações nem no fator de impacto.

**Conclusão:**

Artigos brasileiros são significativamente menos aceitos em revistas de maior impacto sem aparente comprometimento de sua repercussão na comunidade científica.

## INTRODUCTION

The Brazilian scientific production has experienced a vertiginous advance over the last three decades, placing the country among the 20 most productive nations in the world, and the first in Latin America, despite being deficient in quality and impact^(1)^. The areas of health and biology have generated almost 50% of all this production, and Medicine shows one of the most expressive growth rates, producing one fourth of all publications^(2)^. This gain has demonstrated positive reflections on research on cancer, which represent an expressive increase in its publications in the context of historically consolidated areas, such as cardiology and infectious diseases^(3)^. Even so, there are signs that only a small percentage of Brazilian research in the area of oncology is translated into published articles^(4,5)^, which could be the result of its relatively low quality or yet of publication bias, described as a tendency to publish results of studies based on the strength and directions of its findings^(6)^.

Therefore there is a general perception that Brazilian studies on cancer are predominantly published in lower impact factor (IF) journals, which would correspond to a measurement of its prestige. Part of this perception occurs because of the factors that influence publishers in accepting or rejecting articles are not clear; even though the literature is full of assays trying to uncover such a fact ^(7–10)^.

## OBJECTIVE

The present study had the objective of investigating if Brazilian articles on cancer are published in journals with inferior prestige than articles that come from foreign institutions, when compared, in terms of their repercussion, by means of matching according to number of citations. The secondary objective is to evaluate if national articles show repercussion similar to international articles when published in journals with equivalent IF, and if the nationality of the institutional affiliation of the article's first author is correlated to the number of citations and/or to the IF of the journal that accepted it.

## METHODS

During the period from July 1^st^ to 31^st^, 2012, the PubMed database was searched using the MeSH Term “neoplasm”. Limits were defined to filter studies with the following characteristics: clinical trial, author affiliation to Brazilian research institutions and published during the interval of July 1^st^, 2009 to June 30, 2010. Articles were excluded if they dealt with benign neoplasms and/or if they were not related to the theme of oncology/cancerology.

Once the national articles were identified and using as reference their dates of publication, citations from correlated articles were selected for matching (Related Citations in PubMed), automatically available beside the national article on PubMed electronic page (http://www.ncbi.nlm.nih.gov/pubmed). Of these correlated citations, those with non-Brazilian affiliations were selected, which had been published during the interval from 3 months prior to 3 months after the date of publication of the reference Brazilian study. From this selection, articles originated in national institutions that dealt with benign neoplasms or that did not show a relation with the oncology/ cancerology theme were excluded. That set of articles was called related international articles.

Once the national and related international articles had been selected, a new search was carried out, now using the Web-of-Science^®^ (WOS, Thomson & Reuters) and SciVerse^®^ (SC, Scopus) databases, to evaluate the number of citations for each one of the studies selected.

Lastly, the IF of the respective national and international articles was collected. For this, the database Journal Citation Reports^®^ (JCR, Thomson & Reuters) 2011 edition was used.

### Comparative analysis of IF between national and international articles

For evaluation of a possible publication bias, by which national articles would be published in journals with a lower IF, matching was made using national articles and international articles that had a similar number of citations obtained in both databases. Considering the number of citations of the national study, it was determined as adequate for matching a variation of two citations more or less of the international article. Within this limit, two international articles were sought for each national article. When more than two international articles were located, the chosen one(s) was/were the one(s) with the publication date closest to the national article. When only one international article met the criteria, this one was selected. However, when no international study was suitable for matching, the national article was excluded for the effect of this comparison. The group of international articles matched was called “corresponding international articles by number of citations.”

### Comparative analysis of the number of citations between national and international articles

In order to evaluate if there would be less repercussion of national articles, national and international articles were matched according to the IF of their journals. For this, international studies were chosen with a maximal IF of 1.0 point more or less to the corresponding national article. For this matching, four international articles were sought for each national article. When more than four international articles met such a criterion, the one chosen was that with the publication date closest to the national article; if even so more than four studies were compatible with the criteria, all such studies were included in the matching process. When less than four articles met this criterion, all were selected. However, when no international study proved capable of being matched with the national publication, the national article was excluded for this comparison. The group of matched international articles was called “corresponding international articles according to IF.”

### Study affiliation

Affiliation was identified, taking account the country of origin of the first author, for the corresponding international articles according to the number of citations and by IF. Articles originated in England, Ireland, Scotland, and Wales were grouped as the United Kingdom. Posteriorly, were identified the articles from the ten countries that most published in the area of oncology/ cancerology between the years of 1993 and 1999 (TOP 10), based on the study by Grossi et al.^(11)^.

### Statistical analysis

In order to compare the means of numbers of citations and of the impact factors between the groups of national and international articles, the non- parametric Mann-Whitney test was used, since the distribution of the variables was not of normal type. Since the goal was to evaluate if the IFs and the number of citations of national articles would be or not inferior to those of foreign articles matched with them, statistically significant values were considered one-tailed p values inferior to 0.05. For statistical analysis, the VassarStats (www.vassarstats.net) and GraphPad Prism^®^ 5 (www.graphpad.com) software were used.

## RESULTS

During the period evaluated, were identified 55 national articles (29 from the year 2009 and 26 from the year 2010). Twelve articles were excluded (8 from the year 2009 and 4 from the year 2010) since they presented a focus on benign neoplasms or did not address oncology/cancerology. Among the related international articles, 876 met the previously defined criteria (Table 1).

**Table 1:** Matching of national and international articles

Articles selected		n
	National reference articles	43
	Related international articles	876
Comparative analysis of IF between national and international articles		
Matching based on number of citations (ratio 1:2)		
	Selected national articles	32
	Corresponding international articles	58
Comparative analysis of number of citations between national and international articles		
Matching based on IF (ratio 1:4)		
	Selected national articles	30
	Corresponding international articles	95

IF: Impact factor.

As for the journals which the Brazilian articles were published in, four publications still did not show a FI evaluation in the 2011 edition of the JCR. For those with defined IF (39), the mean was 2.734. Among the related international articles, 812 showed publications with a defined IF, with a mean of 3.516. Before matching, no difference was observed between the IF of the national and related international articles (p=0.229) (Table 1). Only seven national articles were published in Brazilian journals; of these, four had a defined IF, with a mean of 0.812, whereas the mean IF of those published in foreign journals was 3.260, showing a significant difference (p<0.0001).

Also, before any matching, the number of citations in the WOS database showed a mean of 4.568 for national articles and 7.476 for related international articles (p=0.131). On the other hand, in the SC database, the mean for the national articles was 6.163 and for the related international articles it was 8.222 (p=0.297) (Table 2). Among the national studies published in Brazilian journals, the mean number of citations was lower (WOS: 1.000; SC: 2.571), but with no statistical significant difference when compared to Brazilian studies published in foreign journals (respectively, p=0.084 and p=0.166).

**Table 2:** Characteristics of selected national articles and related international articles (before matching)

Articles		IF of journals	Number of citations in the database WOS	Number of citations in the database SC
Reference national articles	n	39	37	43
	Mean	2. 734	4,568	6,163
	Range	9.379	26,085	3,633
	Standard deviation	3.062	5,107	6,027
	Median	3.862	10	5
Related international articles	n	812	799	860
	Mean	3.516	7,476	8,222
	Range	15.945	133,876	159,800
	Standard deviation	3.993	11,570	12,641
	Median	2.844	4	5
p value		0.229	0.131	0.297

IF: Impact factor; WOS: Web of Science^®^; SC: SciVerse^®^.

### Comparative analysis of IF and the number of citations between national and international articles

Eleven national articles were excluded from this analysis: 4 for having been published in journals with no defined IF and 7 because no other related international article having been found which fit the interval of citation number previously determined. Thus, for this analysis, from the 32 evaluable national articles only 26 were matched with 2 international articles and 6 with only one corresponding international study, summing up 58 corresponding international articles by number of citations (Table 1).

**Figure 1:**
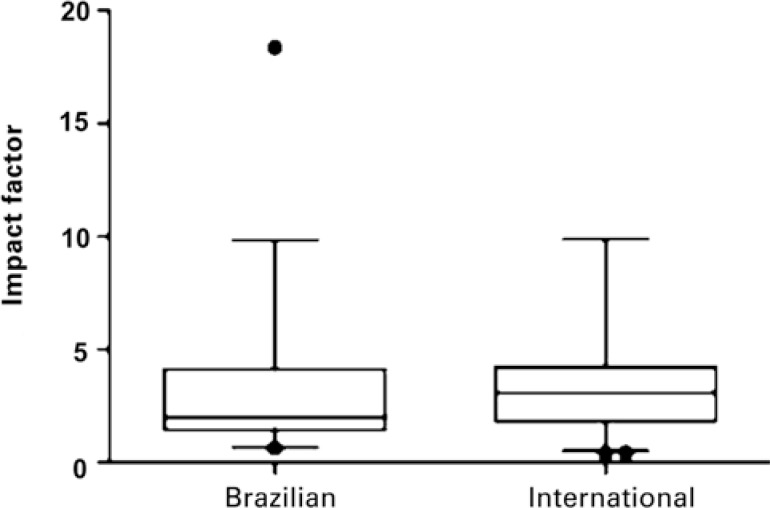
Impact factor of journals in which selected Brazilian and international articles were published (p=0.041), after matching per number of citations

After matching by number of citations, the mean of IF for Brazilian articles (32) was 3.000; conversely, for the corresponding international articles by number of citations (58), it was 3.430. Considering one-tailed p, a relevant difference was observed (p=0.041) (Figure 1).

By matching Brazilian with international studies according to the IF, only 30 national studies were included: 4 were excluded for not having a defined IF, and 9 for not having corresponding international articles. The 30 national articles ended up being matched with a total of 95 corresponding international articles by IF (Table 1). No difference was observed for the number of citations among the national and corresponding international studies, considering the two databases studied (WOS: p=0.201; SC: p=0.33) (Table 3).

**Table 3:** Number of citations among national and corresponding international articles matched by impact factor

Database		National articles	International articles	p value
Web of Science^®^	n	29	90	
	Mean	5,793	4,725	
	Range	34,384	23,779	0.201
	Standard deviation	5,864	4,876	
SciVerse^®^	n	30	91	
	Mean	6,667	5,630	
	Range	42,299	29,928	0.333
	Standard deviation	6,504	5,471	

### Correlation between the number of citations and the IF of national and international articles

**Figure 2:**
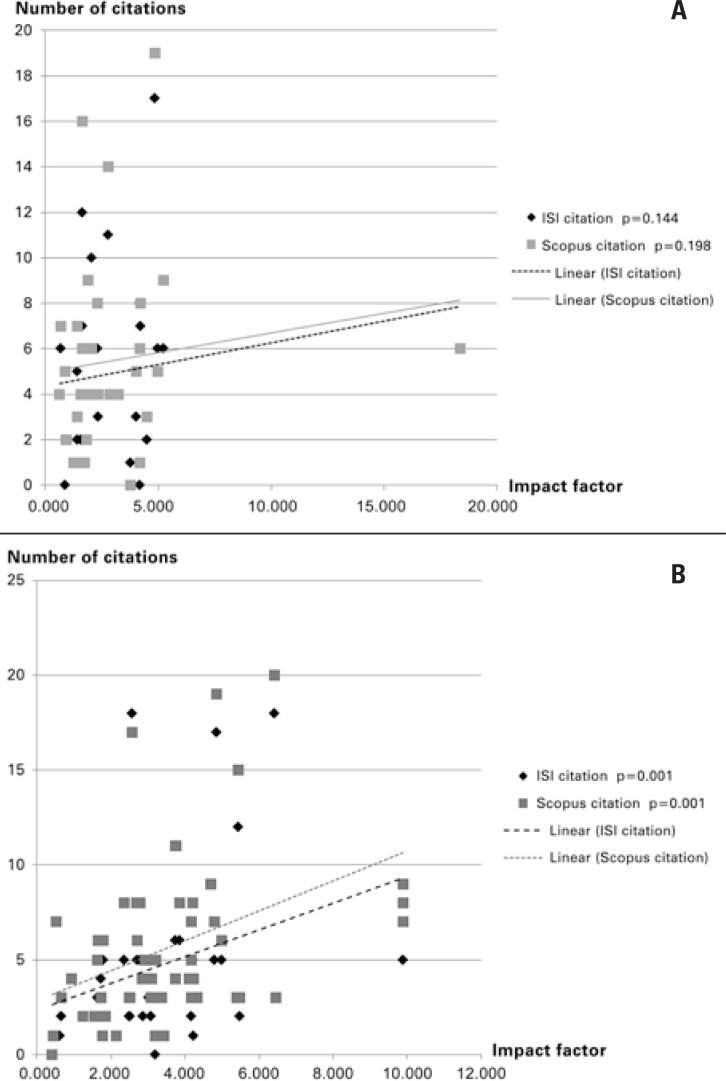
**A)** Correlation between number of citations and IF of journals for national articles (Web of Science^®^: p=0.144; SciVerse^®^: p=0.198). **(B)** Correlation between number of citations and IF of journals for international articles (Web of Science^®^: p=0.001; SciVerse^®^: p=0.001)

Considering all national reference articles (43) and their related international articles (876), the number of citations was correlated based on the IF of the journals. Among the national studies, no significant correlation was observed between IF and the number of citations, although there is a positive tendency (Figure 2A). On the other hand, for the related international articles, the significant correlation between IFs and the number of citations is clear (Figure 2B).

### Evaluation of the corresponding international articles

For all 58 corresponding international articles by number of citations, the country of origin was found. Considering the number of corresponding articles, the United States was the most representative country (29.31%), followed by Italy and China (8.62%, each) and the United Kingdom (6.90%). Whereas, when taking into consideration the mean IF of the articles, Belgium is the first (7.059), followed by India (6.452) and by Holland (5.771) – although the second had only 1 article represented (Table 4).

**Table 4:** Affiliation and impact factor of corresponding international articles

Country	Number of articles n (%)	Total IF	Mean IF	Median IF
United States	17 (29.31)	59.614	3.507	3.160
Italy	5 (8.62)	17.460	3.492	2.685
China	5 (8.62)	13.900	2.780	2.780
United Kingdom	4 (6.90)	11.828	2.957	3.861
Germany	3 (5.17)	15.140	5.047	4.991
France	3 (5.17)	4.673	1.558	2.132
Belgium	2 (3.45)	14.117	7.059	7.059
Holland	2 (3.45)	11.541	5.771	5.771
Portugal	2 (3.45)	8.113	4.057	4.057
Switzerland	2 (3.45)	6.379	3.190	3.190
Austria	2 (3.45)	5.384	2.692	2.692
Japan	2 (3.45)	1.586	0.793	0.793
India	1 (1.72)	6.452	6.452	6.452
Spain	1 (1.72)	4.238	4.238	4.238
Australia	1 (1.72)	4.182	4.182	4.182
Canada	1 (1.72)	4.182	4.182	4.182
South Korea	1 (1.72)	3.746	3.746	3.746
Rumania	1 (1.72)	1.847	1.847	1.847
Slovenia	1 (1.72)	1.551	1.551	1.551
Malaysia	1 (1.72)	1.240	1.240	1.240
Croatia	1 (1.72)	0.614	0.614	0.614

IF: Impact factor.

Thirty-eight corresponding international articles by number of citations met the criteria for the TOP 10. Comparing the IF values of these studies with the reference national articles (32), no significant difference was observed (p=0.517) (Table 5).

**Table 5:** Evaluation of impact factor and number of citations of selected national articles *versus* corresponding international articles included in TOP 10, according to Grossi et al.^(11)^

Citations		Reference national articles	TOP 10 international articles	p value
IF	n	32	38	
	Total	95.996	130.262	
	Mean	3.000	3.430	0.517
Number of citations in WOS	n	32	67	
	Total	178	341	
	Mean	5,933	5,090	0.473
	n	32	67	
Number of citations in SC	Total	200	407	
	Mean	6,668	6,075	0.647

IF: Impact factor; WOS: Web of Science^®^; SC: SciVerse^®^.

When evaluating the 95 corresponding international articles by IF, it was noted that 67 of them were a part of the TOP 10. Comparisons were made for the number of citations between the national reference articles and these 67 studies. For the two databases studied, no significant difference was found (WOS: p=0.473; SC: p=0.647).

## DISCUSSION

Despite the increased number of Brazilian publications on cancer, in pace with the growth of national scientific production, the country still shows difficulty in exposing its initiatives in publications of greater visibility. In fact, the present study revealed that, in comparing studies with similar scientific repercussion, judging by the equivalent number of citations, the Brazilians are accepted in publications with a mean IF of 3.000, while the international articles are published in journals with a mean IF of 3.430. This difference is statistically significant (p=0.041) (Figure 1). Such a difference becomes slightly more significant (p=0.036) if one of the Brazilian articles that has a publication IF of 18.372, very distant from the group mean, is excluded from the analysis.

The progress in research in the area of oncology has been more pronounced that areas previously consolidated in the Brazilian scientific community, such as cardiology and research on malaria^(3)^. There is still a clear increase of Brazil's participation in the two main world forums for the exposure of advances in cancer research: American Society of Clinical Oncology (ASCO) and European Society of Medical Oncology (ESMO)^(5)^. However, up until then, there were no signs that these advances had had a reflection on ranking in the international scientific community, so that Brazil does not figure in among the countries that publish the most, representing less than 0.5% of the publications on cancer in the world^(3,5,11)^. In fact, there seems to be a gap between the number of active research studies and those that, in the end, translate into publications. An example of this is that, along with countries such as Turkey, South Korea, and Spain, Brazil contributes more with abstracts in congresses than with publications in the main international journals about oncology^(5)^. A study by Saad et al.^(4)^ corroborates this finding in revealing that only 16.9% of the abstracts published at ASCO, between 2001 and 2005, ended up being published in indexed journals. The reasons implied for this phenomenon are innumerable and, among them, are probably limitations of development, the language barrier, and the quality of Brazilian studies. Such reasons, however, can not only impede the leap between a congress abstract to an article published in a journal indexed in international databases, but also, if it ends up being published, this may occur in a publication of lower prestige, as the present study revealed.

The results described here are subject to various limitations. The first of them is the use of the IF as quality indicator of the publication studied. Since the 1960's, when it was created^(12)^, the IF has been used as the most popular indicator to evaluate the quality of a publication, since it represents a picture of the visibility of the articles it contains. Nevertheless, it does not represent an individual article, and depends on the field of research in which the publication is inserted and on the current interests of the researchers^(13,14)^. Even so, this parameter was chosen since it is easily accessible, broadly used by the world scientific community, and allows the comparison with prior studies on the subject in question.

Another limitation is the use of the number of citations as an isolated factor to infer the repercussion of a given article in the scientific community. We point out, however, that this is recognizably an objective measure of individual impact^(15)^ and it has already been said that, in citing an article, the researcher demonstrates that this study exerted influence on him/her in some way, thus reflecting, beyond its repercussion, also its credibility and quality^(16)^. In the present study, it was also considered that the international articles compared with the Brazilian ones should be related, meaning, should present a spectrum of equivalent investigation (according to the automatic selection of related articles by PubMed website), as well as, similar time of publication, sufficient for it to have had the same chance of having been cited, which allowed an analysis between Brazilian and international studies with very similar editorial profiles.

In face of the concept that a relevant article is, therefore, the one which is cited often, arises the principle that publishing in a journal with a high IF will positively influence the number of citations^(17)^. This thesis was put to the test by two methods in the present study. The first method, the number of citations was correlated with the IF of the publications, considering all the reference national articles and their respective related international articles. For the international articles there was a clear and significant correlation between the two parameters for the two databases (WOS: p=0.001; SC: p=0.001); for the national articles, this correlation was not significant (WOS: p=0.144; SC: p=0.198), although there was a clearly positive graphic tendency (Figure 2A). In a second method, the articles were matched based on the IF of their publications, and then the number of citations between them was evaluated, demonstrating the absence of any significant statistical difference between the Brazilian and international studies for both databases studied (WOS: p=0.201; SC: p=0.333). This last result allows inferring that national research on cancer has the same chance of being cited as its international correspondents, when published in journals with equivalent visibility.

These results are compatible with prior findings, which demonstrated that the IF of a publication is the primary predictor of citations of an article. This leads one to believe that an important or seminal article submitted to a journal of lower impact may not receive the recognition that it deserves, as well as a weak article published in a higher impact journal may receive recognition beyond what it deserves^(18)^. Even the individual prestige of a researcher, based on the number of citations that his/her articles have, does not seem to be able to increase the repercussion of an article, when this is published in journals with a lower IF^(19)^.

Despite this, there are signs that other factors, such as the degree of development of a country^(10)^ and the simple geographic origin of it, may directly influence the repercussion of the study. An article by Meneghini et al. demonstrated that, in evaluating seven high IF publications, the articles with exclusively Brazilian authors showed a number of citations significantly lower than international collaborative studies^(20)^. The results presented here contradict these data, not only in demonstrating that the number of citations of Brazilian articles is not different from that of international studies, but also because out of the 43 national articles selected, only two were international and multicenter, and even so, no difference was evident as to the number of citations. Even when confronted with the articles published by more representative countries in the international scientific community (TOP 10), no significant difference was observed (WOS: p=0.473; SC: p=0.647). It is possible, therefore, that within the strict universe of the studies on cancer, the origin of the authors exerts a smaller influence on the repercussion of an article. However, it is not improbable that, knowing the negative impact related to the origin of the article, publishers of higher impact journals might reject those sent from certain countries, as has been suggested before^(8,17,21)^.

Another result to be considered is that, among the 43 national articles selected, only seven were published in Brazilian journals; of these, four already had defined IFs, with a mean of 0.812. On the other hand, the mean IF of those published in foreign journals was 3.260, revealing a significant difference (p<0.0001). This great distance between the IFs seems to be a reflection of the recent inclusion of Brazilian journals in international indexers. Up until now, there are still less than 20 of those with an IF greater than 1.000^(22)^. It must be noted, as well, that the results reveal a clear preference for foreign publications, which without a doubt, has as one of the primary motives the simple fact of not existing, to date, any Brazilian journal dedicated to the research of cancer indexed in international databases and that it is known that the area of oncology is extremely influenced by North-American and European parameters, which may also suggest the choice of their journals. Even so, it is necessary to point out that it is a “cultural” practice to try to publish exclusively in foreign journals for reasons that go from the recognized merit until the incentive from the financing institutions^(23)^.

## CONCLUSIONS

Considering the concept of publication bias in a restricted manner, up until now, it is not possible to affirm that Brazilian articles on cancer have suffered a publication bias. Nevertheless, the findings confirm the general perception that the articles of the country are less accepted in journals of higher IF. Even so, such a fact does not seem to be associated with the quality of the studies, since when compared to international equivalents, no differences were observed in the repercussion of their data, even when confronted with the countries that most publish in the world. It is also relevant to point out the low level of participation of national journals among those chosen by Brazilian researchers to expose their results. These are the data known so far from the first comparison of quality indicators among Brazilian articles published on cancer and the international scenario. Such results may be taken into account so that investigators might be dedicated to expand the Brazilian editorial participation in worldwide and national oncology, and especially, in valuing the national journals, thus reducing the editorial barriers imposed by foreign publications.
